# Supplementary material for: Large-Area Film Thickness Identification of Transparent Glass by Hyperspectral Imaging
Source: Sensors (Basel). 2024 Aug 6;24(16):5094. doi: 10.3390/s24165094 (PMC11359249; doi:10.3390/s24165094)
Supplement: Supplementary file 1 [file sensors-24-05094-s001.zip › sensors-3072840-supplementary-REVISED.pdf]

sensors

Supplementary Materials for

Large-Area Film Thickness Identification of Transparent Glass by Hyperspectral Imaging

Shuan-Yu Huang [1], Riya Karmakar [2], Yu-Yang Chen [2], Wei-Chin Hung [3], Arvind Mukundan [2,*] and Hsiang-Chen Wang [2,4,*]

1 Department of Optometry, Central Taiwan University of Science and Technology, Taichung City 40601, Taiwan; 108695@ctust.edu.tw
2 Department of Mechanical Engineering, National Chung Cheng University, Min Hsiung 62102, Taiwan; karmakarriya345@gmail.com (R.K.); a15937a5566@gmail.com (Y.-Y.C.)
3 Department of Physics, R. O. C. Military Academy, Kaohsiung City 830208, Taiwan; hung.wc0602@msa.hinet.net
4 Director of Technology Development, Hitspectra Intelligent Technology Co., Ltd., Kaohsiung 80661, Taiwan
* Correspondence: d09420003@ccu.edu.tw (A.M.); hcwang@ccu.edu.tw (H.-C.W.)

Abstract: This article gives the supplementary information for the article "Large-Area Film Thickness Identification of Transparent Glass by Hyperspectral Imaging". The first section provides the individual conversion formulae used for the VIS-HSI conversion algorithm. Section 2 provides the individual conversion formulae for the NIR-HSI conversion algorithm.

Keywords: hyperspectral imaging; near-infrared; transparent glass; thickness estimation

S1. Visible Hyperspectral Imaging

The individual conversion formulas to convert the 24-color patch image and 24 color patch reflectance spectrum data to XYZ color space are as follows

On the camera side: convert sRGB color gamut space to XYZ color gamut space

$$\begin{bmatrix} X \\ Y \\ Z \end{bmatrix} = [M_A][T]\begin{bmatrix} f(R_{sRGB}) \\ f(G_{sRGB}) \\ f(B_{sRGB}) \end{bmatrix} \times 100\,, 0 \le \begin{matrix} R_{sRGB} \\ G_{sRGB} \\ B_{sRGB} \end{matrix} \le 1 \tag{S1}$$

where

$$[T] = \begin{bmatrix} 0.4104 & 0.3576 & 0.1805 \\ 0.2126 & 0.7152 & 0.0722 \\ 0.0193 & 0.1192 & 0.9505 \end{bmatrix} \tag{S2}$$

$$f(n) = \begin{cases} (\frac{n+0.055}{1.055})^{2.4}, n > 0.04045 \\ \left(\frac{n}{12.92}\right), otherwise \end{cases} \tag{S3}$$

$$[M_A] = \begin{bmatrix} X_{SW}/X_{CW} & 0 & 0 \\ 0 & Y_{SW}/Y_{CW} & 0 \\ 0 & 0 & Z_{SW}/Z_{CW} \end{bmatrix} \tag{S4}$$

On the spectrometer side: convert reflection spectral data to XYZ color gamut space

$$X = k\int_{400nm}^{700nm} S(\lambda)R(\lambda)\bar{x}(\lambda)d\lambda \tag{S5}$$

$$Y = k\int_{400nm}^{700nm} S(\lambda)R(\lambda)\bar{y}(\lambda)d\lambda \tag{S6}$$

$$Z = k\int_{400nm}^{700nm} S(\lambda)R(\lambda)\bar{z}(\lambda)d\lambda \tag{S7}$$

$$k = 100/\int_{400nm}^{700nm} S(\lambda)\bar{y}(\lambda)d\lambda \tag{S8}$$

The nonlinear response of the camera can be corrected by a third-order equation, and the nonlinear response correction variable is defined as $V_{Non\text{-}linear}$.

$$V_{Non-linear} = [X^3\ Y^3\ Z^3\ X^2\ Y^2\ Y^2\ X\ Y\ Z\ 1]^T \quad \text{(S9)}$$

In the dark current part of the camera, the dark current is usually a fixed value and does not change with the amount of incoming light, so a constant is given as the contribution of the dark current, and the dark current correction variable is defined as V_{Dark}.

$$V_{Dark} = [a] \quad \text{(S10)}$$

Finally, VColor is used as the base, and multiplied by the nonlinear response correction of $V_{Non\text{-}linear}$, and the result is standardized within the third order to avoid excessive correction, and finally V_{Dark} is added to obtain the variable matrix V.

$$V_{Color} = [XYZ\ XY\ XZ\ YZ\ X\ Y\ Z]^T \quad \text{(S11)}$$

$$V = [X^3\ Y^3\ Z^3\ X^2Y\ X^2Z\ Y^2Z\ XY^2\ XZ^2\ YZ^2\ XYZ\ X^2\ Y^2\ Y^2\ XY\ XZ\ YZ\ X\ Y\ Z\ a]^T \quad \text{(S12)}$$

Before using CIE DE2000 to calculate color difference, $XYZ_{Correct}$ and $XYZ_{Spectrum}$ must be converted from XYZ color space to lab color space. The conversion formula is as follows:

$$L^* = 116f\left(\frac{Y}{Y_n}\right)-16$$
$$a^* = 500\left[f\left(\frac{X}{X_n}\right)-f\left(\frac{Y}{Y_n}\right)\right] \quad \text{(S13)}$$
$$b^* = 200\left[f\left(\frac{Y}{Y_n}\right)-f\left(\frac{Z}{Z_n}\right)\right]$$

$$f(n) = \begin{cases} n^{\frac{1}{3}},\ n > 0.008856 \\ 7.787n + 0.137931, otherwise \end{cases} \quad \text{(S14)}$$

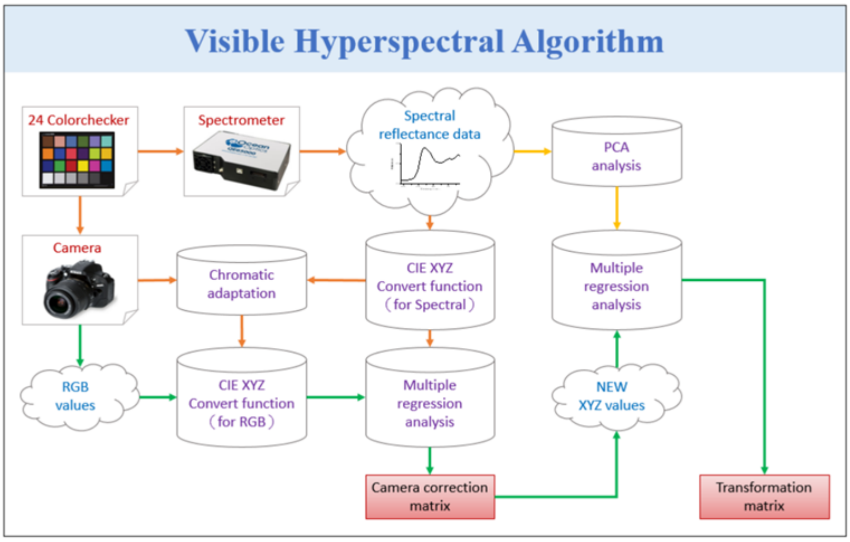

Figure S1. Schematics of the VIS-HSI algorithm.

S2. Near infra-red hyperspectral algorithm

NIR hyperspectral technology can assign the grayscale values measured by the NIR camera to the spectrum information. The radiation energy received in this band is primarily composed of the energy of the substance reflecting the sun. Hence, 14 different substances were selected as reference objects. These substances were passed through halogen lamps and liquid crystal tunable filters to obtain the reflection spectrum of different materials, the grayscale values of the entire band, and the reflection spectrum data of the narrow band to build the NIR hyperspectral technology.

The image obtained by the IR camera is a grayscale image (TIFF 16bit) which can be obtained by installing a liquid crystal adjustable filter in front of the camera to obtain the full- and narrow-band grey values. After obtaining the reflection spectrum of the target, principal component analysis is performed on these spectra, and the six most important principal components are taken. Out of these six principal components eigenvalues and eigenvector can explain 99.79% data variation. Then the corresponding principal

component scores (Score, eigenvalue) are obtained through these six groups of principal components and are used for regression analysis with the grayscale values of the entire band.

In the multivariate regression analysis of the grayscale values and score, the first-order linear equation is used as the variable because this study is based on the change in reflectance with the different materials, and the grayscale values can be regarded as the total integral value of the narrow band data, namely, the average reflectance in this band. Therefore, the value and the first principal component score should be linearly proportional, while the second to sixth principal components are correction terms.

The regression analysis is shown in Equation S14, while the analogy spectrum is shown in Equation S15 ($S_{Spectrum}$). Finally, the root mean square error (RMSE) of each target can be calculated by comparing the obtained analog and measured reflection spectra. The average error was 0.06.

$$[M] = [Score] \times pinv([Grayscalevaluesa] \qquad (S15)$$

$$[S_{Spectrum}]_{(900\sim1700nm)} = [EV] \times [M] \times [Gray\ scale\ values\ a] \qquad (S16)$$

The NIR hyperspectral technology built by the above process can simulate the grey value captured by the NIR camera to the reflection spectrum. If the grey value of the entire image is calculated through the NIR hyperspectral technology, then the NIR hyperspectral image can be obtained by using the above calculations. The algorithm construction process is shown in Figure S2.

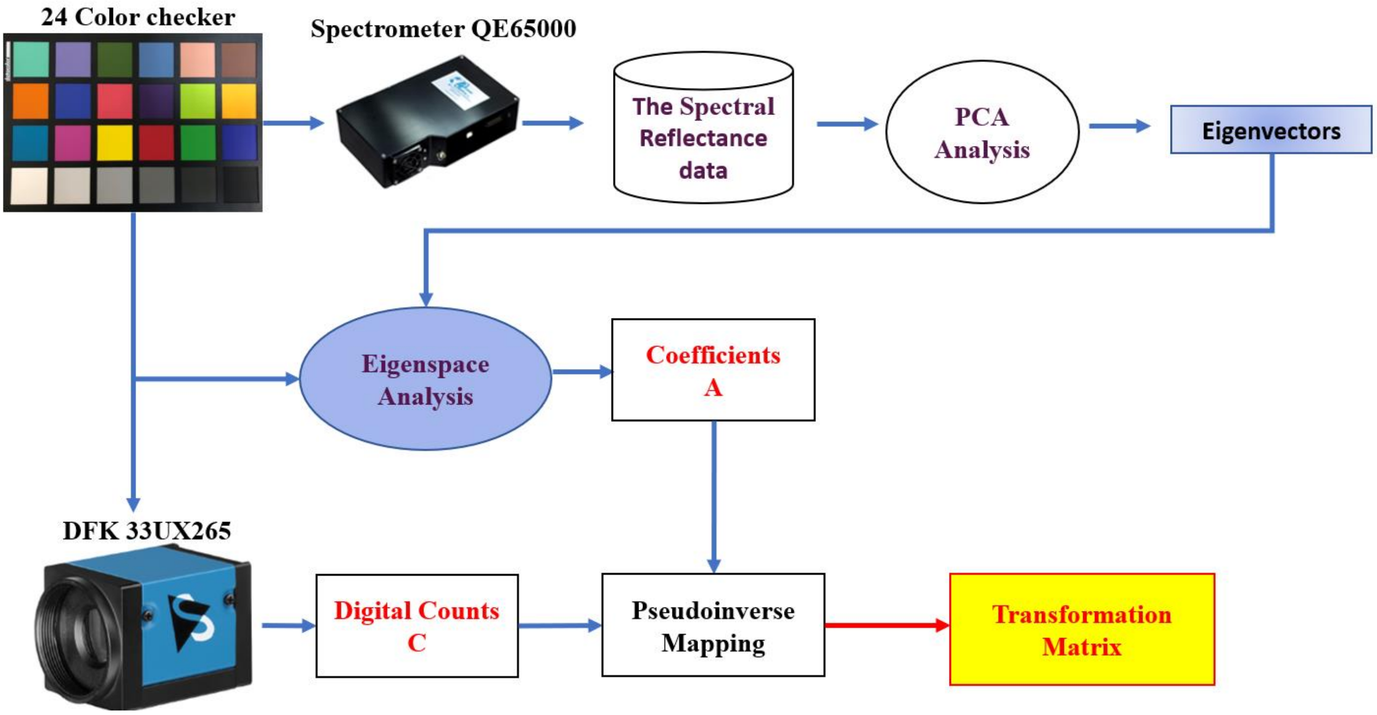

Figure S2. Near-infrared hyperspectral technology construction process.

S3. KLA-Tencor D300 Profiler

The KLA-Tencor D300 Profiler is employed for obtaining surface profile information through the scanning of object surfaces with a sharp-tipped probe as shown in Figure S4. The probe's scanning trajectory traverses the object's surface, while a conductive sensor records the variations in the probe's vertical motion. Capable of measuring two-dimensional step heights ranging from a few nanometers to 1200 mm, the profiler enables the assessment of height measurements in compliance with the surface's elevation differences. This allows for the calculation of glass film thickness based on the probe's motion, serving as the basis for comparing and evaluating accuracy against experimental results.

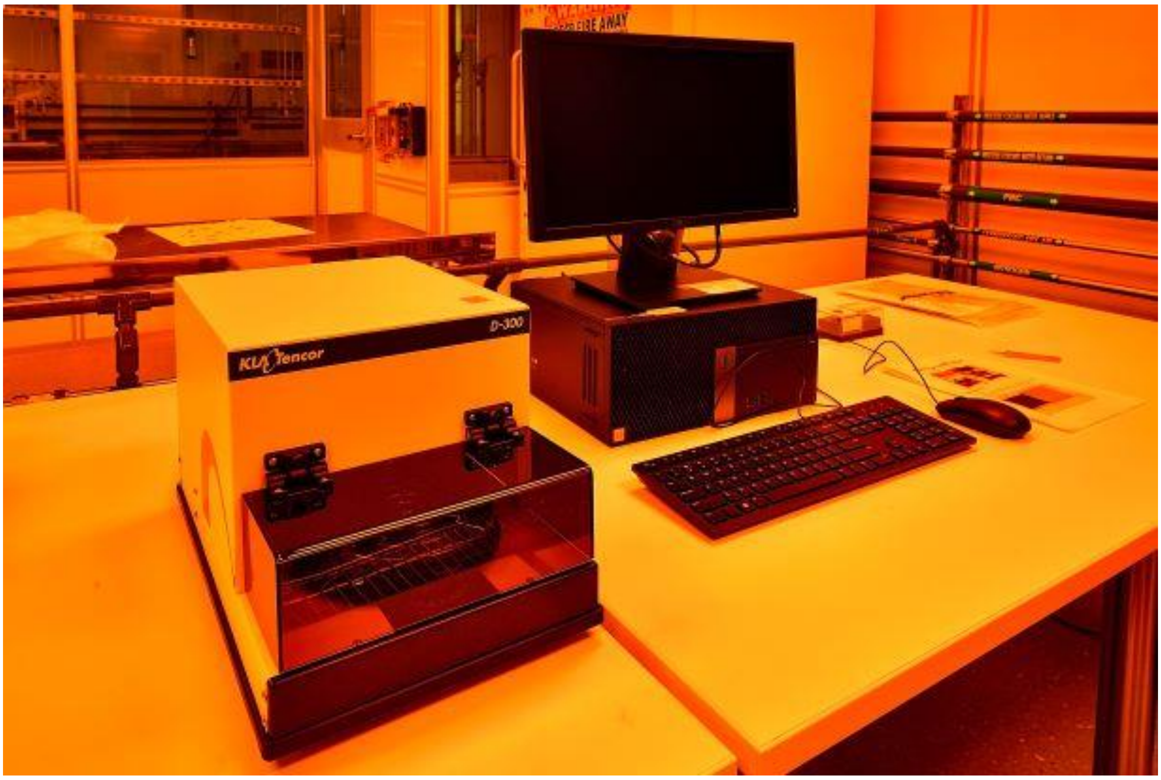

Figure S3. KLA-Tencor D300 Profiler.

S4. A dual-beam spectrophotometer

A dual-beam spectrophotometer, CT-8600, was employed for the comprehensive spectral analysis of the six distinct Low-E glass samples' transmittances. The measurements were conducted at a resolution of 1nm. As the characteristics of glass entail complete transparency within the visible light range (380nm780nm), rendering it indistinctive, no reference value could be established within this band. However, in the near-infrared spectrum (781nm~1100nm), significant variations in transmittance were observed among the experimental samples of differing thicknesses. The transmittance in the near-infrared spectrum was subsequently averaged.

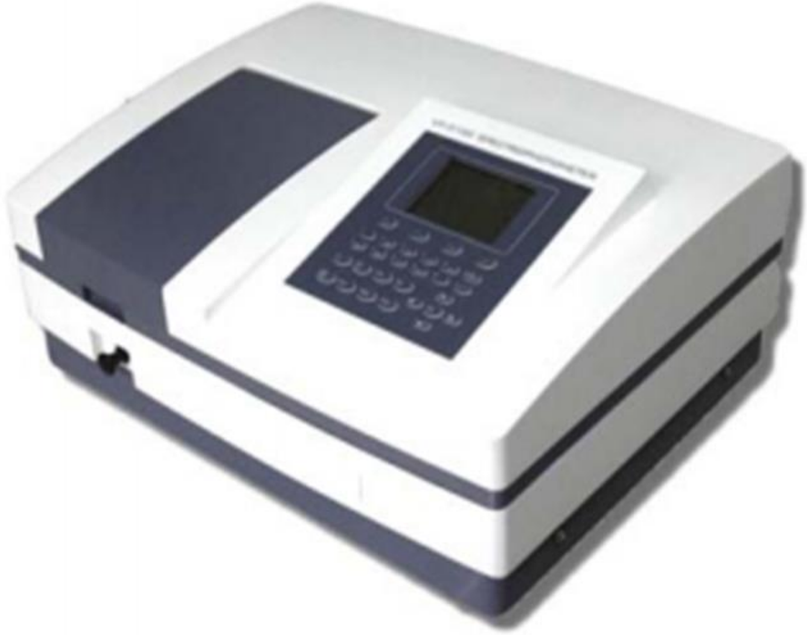

Figure S4. CT-8600 dual-beam spectrophotometer.

S5. Optical Microscope (OM)

An optical microscope (OM), utilizes optical lenses to achieve image magnification. Incident light from the object passes through the optical system of the objective and eyepiece to provide amplification. The nosepiece on the microscope accommodates interchangeable magnifications of 5X, 10X, 20X, 40X, and 100X as required. Depending on the design of the condenser and objective, optical microscopes are categorized into reflective and transmissive types. Reflective microscopes are typically used for non-transparent samples; light is projected onto the object from above, and the reflected light is captured to form the image. They are commonly applied in observing solids, materials, etc. Transmissive microscopes, on the other hand, are designed for transparent or extremely thin samples, enabling light to pass through the sample to produce an image. These are commonly used for observing biological tissues. The stage holds the sample and can adjust the aperture size underneath. In dim light conditions, a larger aperture can be chosen for enhanced illumination. In this study, transmissive microscopy was employed to collect images of glass films. A halogen lamp was used as the lower light source to generate full-spectrum signals. The images were captured at a resolution of 800X600 pixels, as illustrated in Figures S5 to S9. Six experimental samples were documented with their processing parameters, microscope magnification, and capture positions, serving as the data source. The OM used is shown in Figure S10.

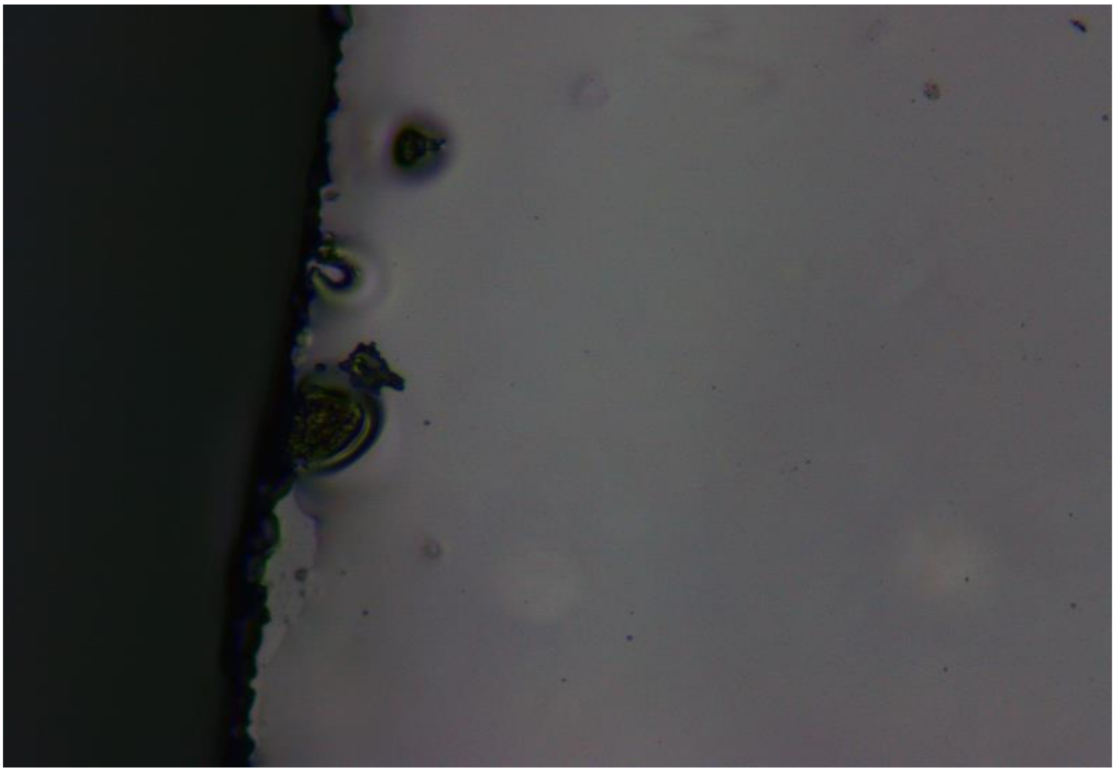

Figure S5. G4000 Microscope image.

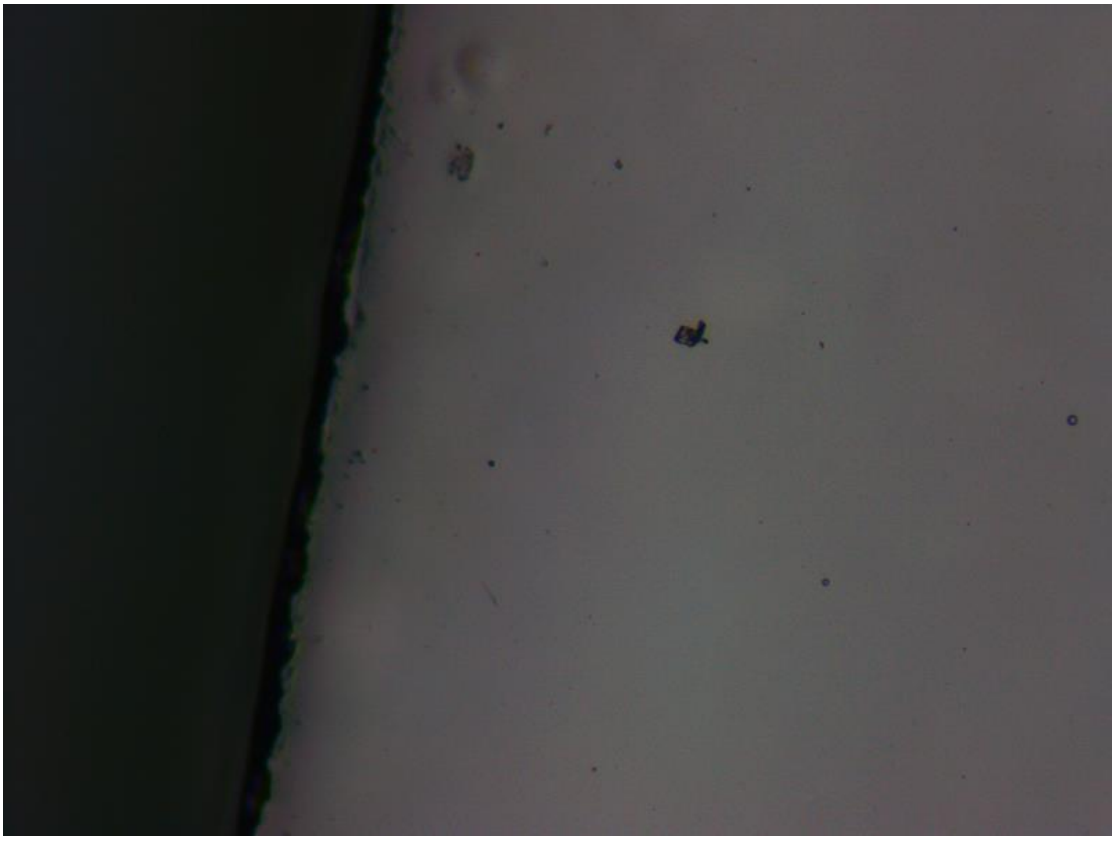

Figure S6. G5000 Microscope image.

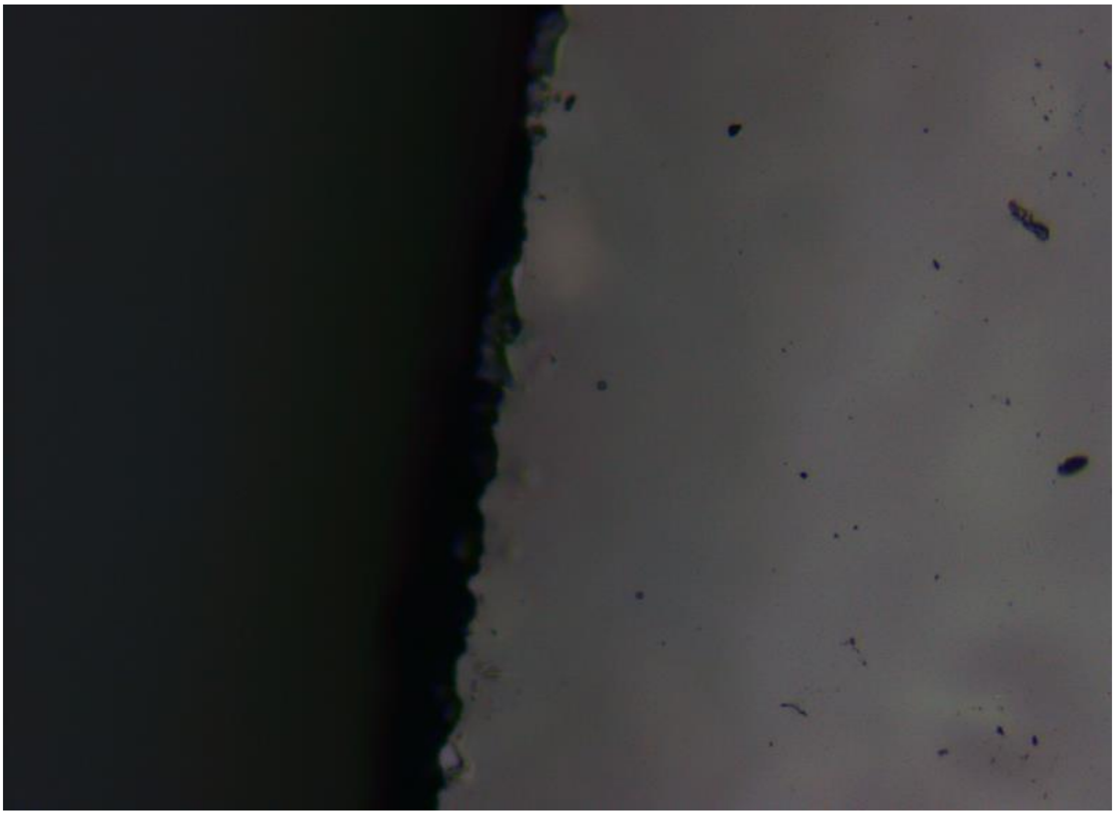

Figure S7. G6000 Microscope image.

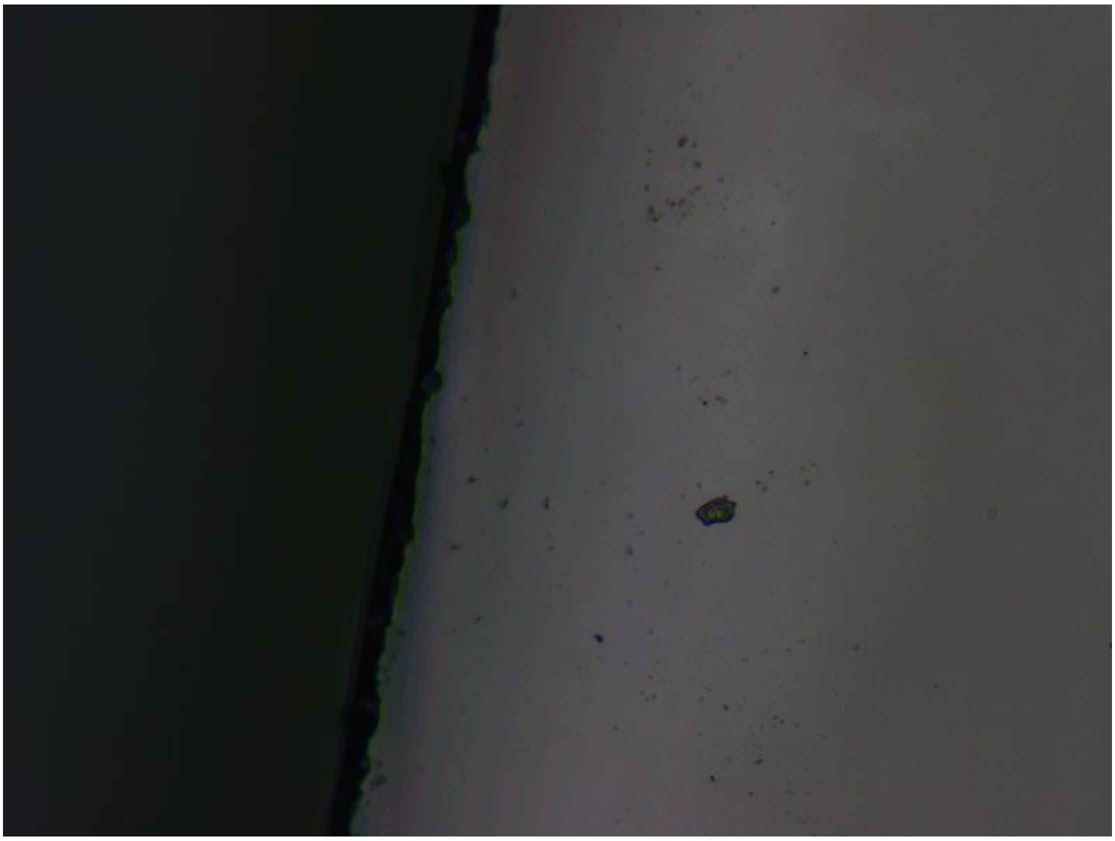
Figure S8. G7000 Microscope image.

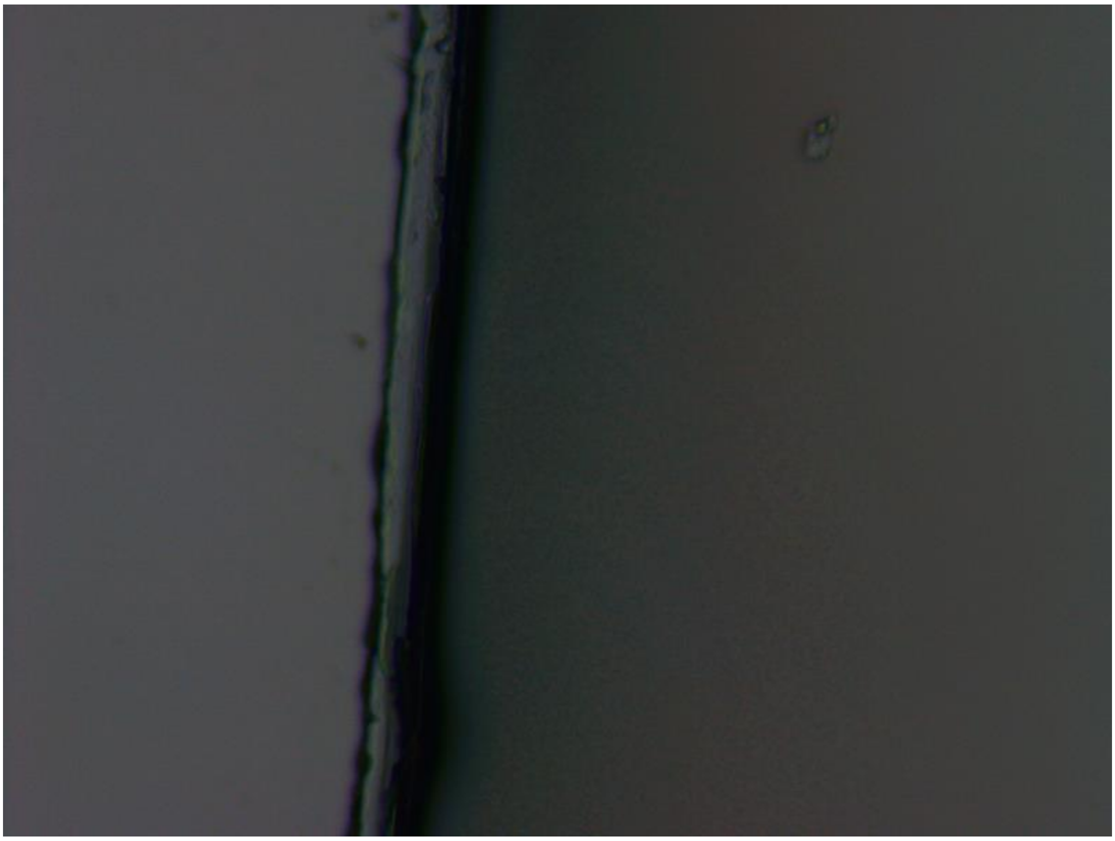
Figure S9. G12000 Microscope image.

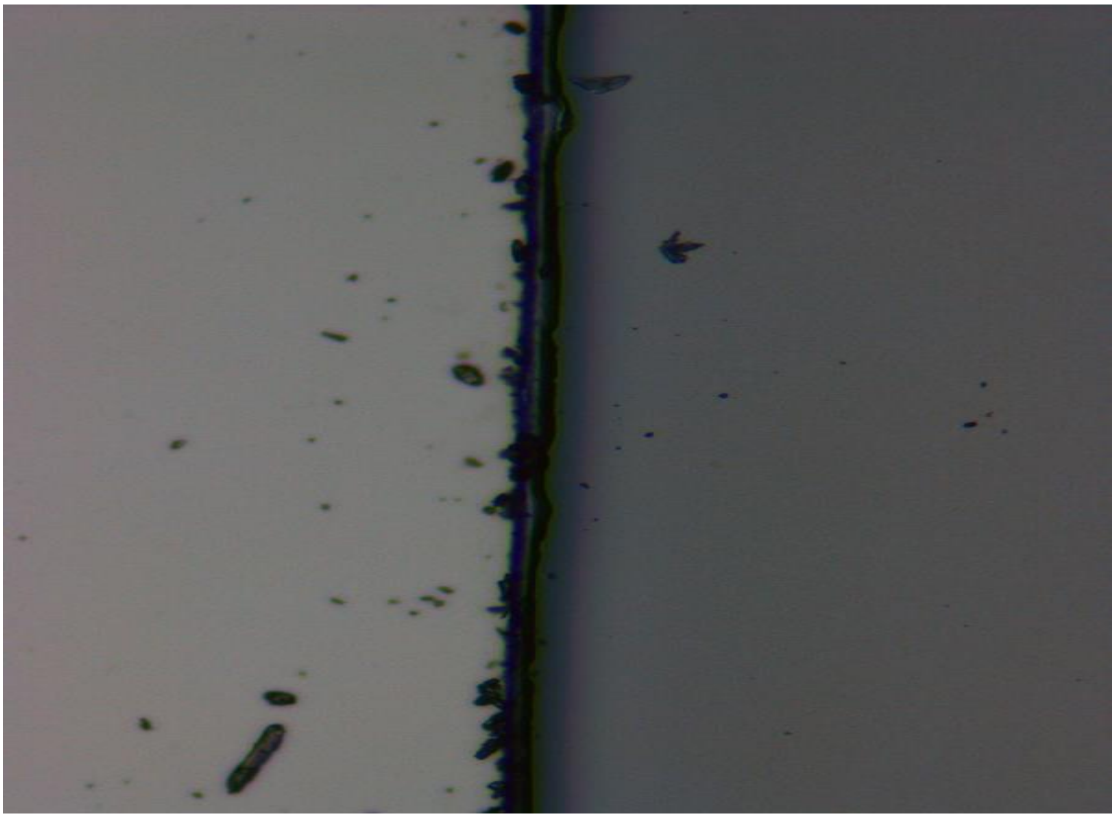

Figure S10. G14000 Microscope image.

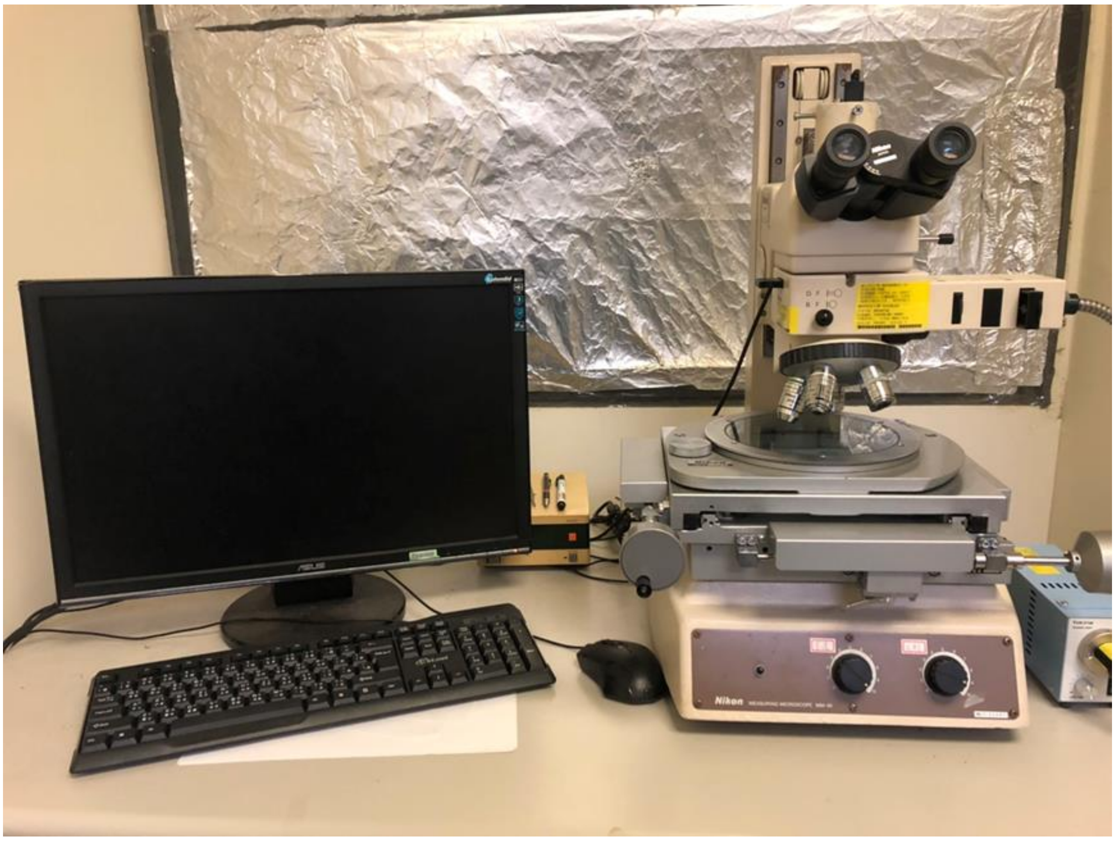

Figure S11. Optical Microscope.

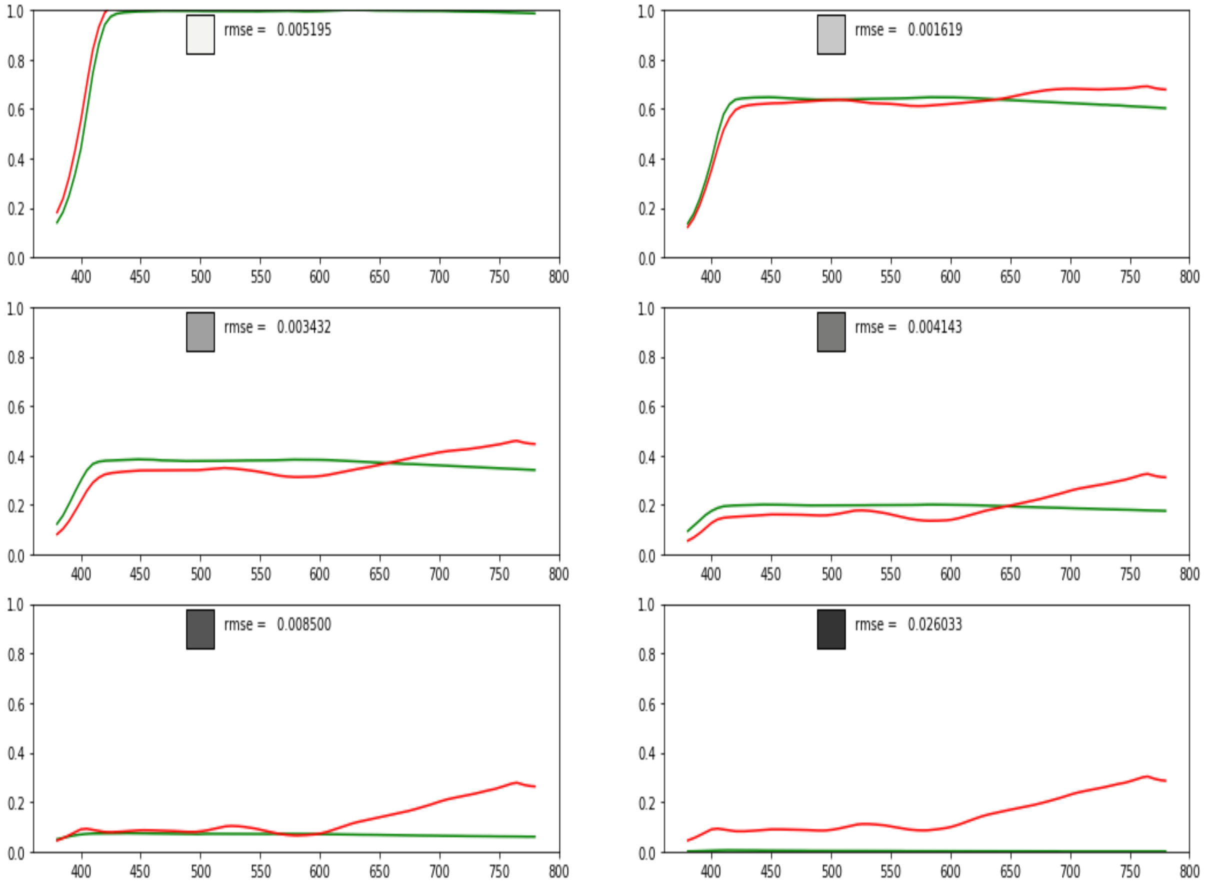
rmse = 0.005195
rmse = 0.001619
rmse = 0.003432
rmse = 0.004143
rmse = 0.008500
rmse = 0.026033

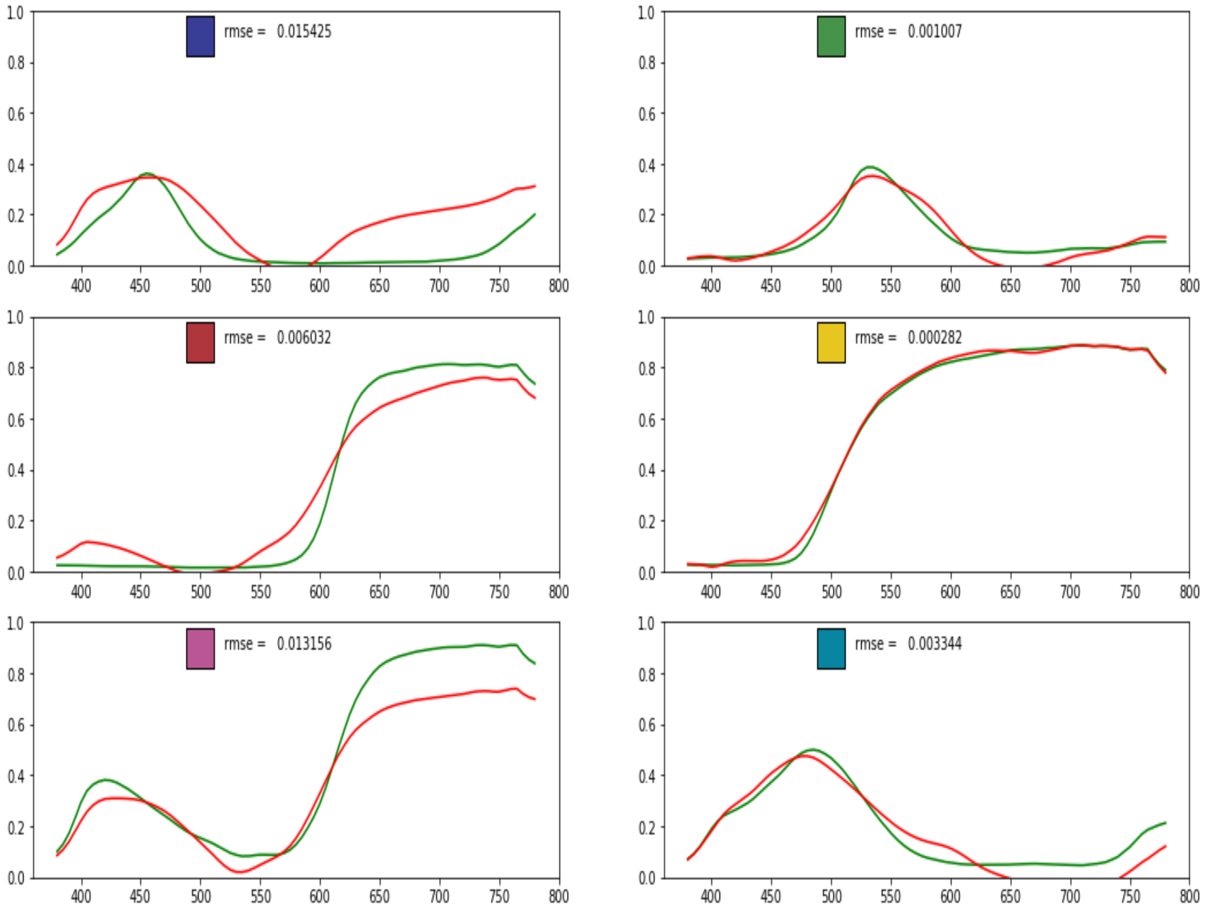
rmse = 0.015425
rmse = 0.001007
rmse = 0.006032
rmse = 0.000282
rmse = 0.013156
rmse = 0.003344

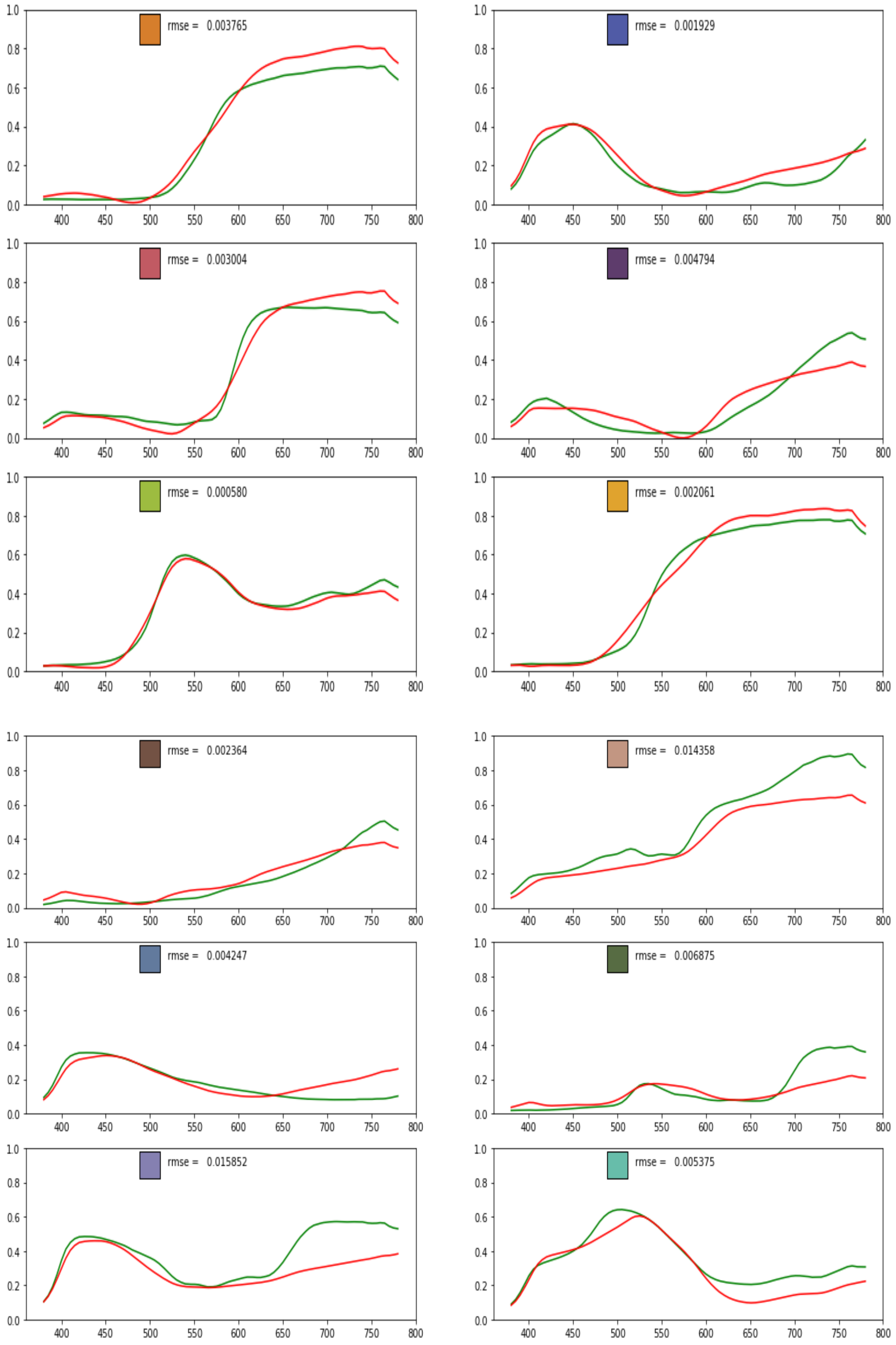

Figure S12. Visible light simulated spectrum (green) and measured spectrum (red).

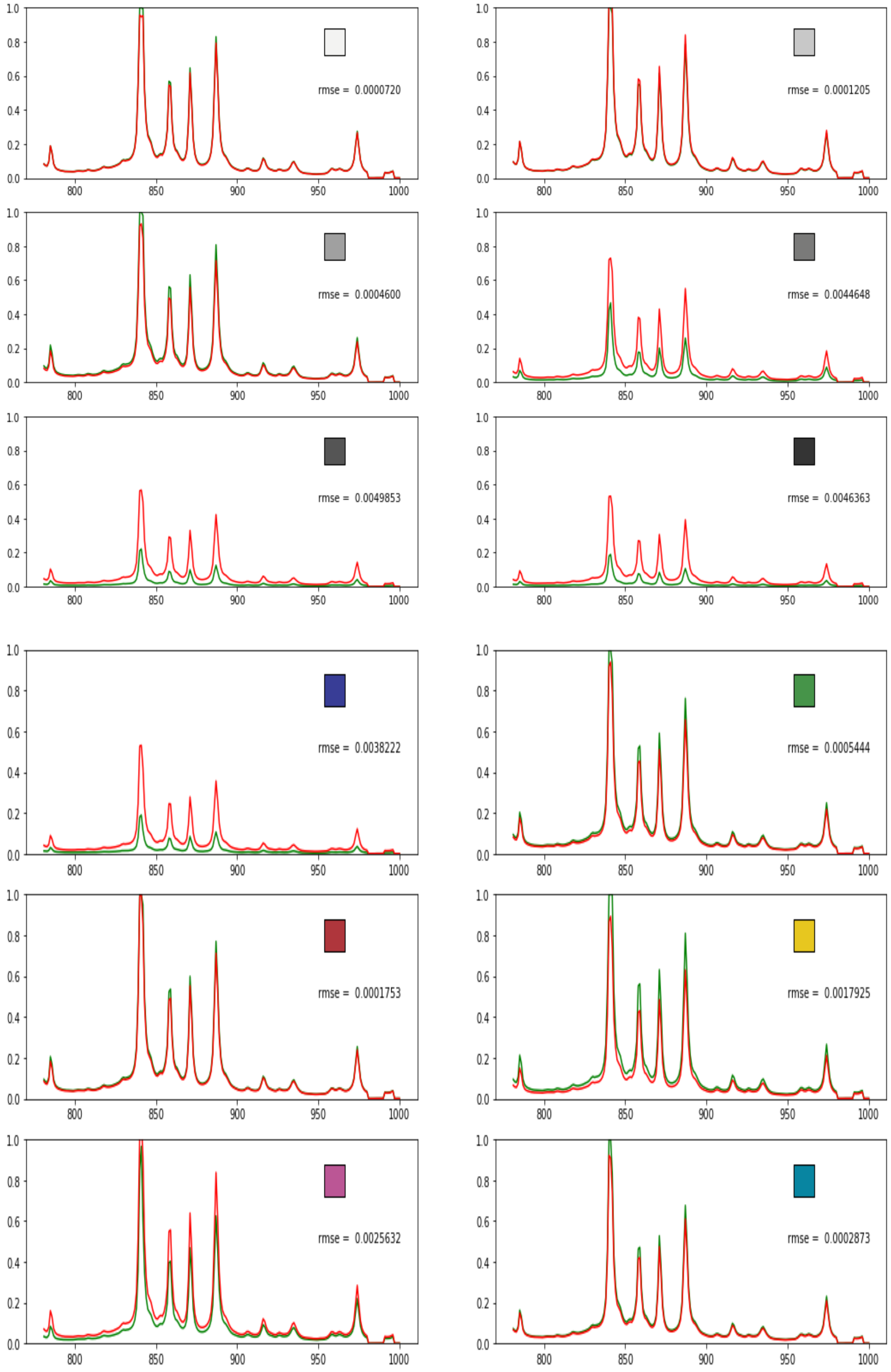
rmse = 0.0000720
rmse = 0.0001205
rmse = 0.0004600
rmse = 0.0044648
rmse = 0.0049853
rmse = 0.0046363
rmse = 0.0038222
rmse = 0.0005444
rmse = 0.0001753
rmse = 0.0017925
rmse = 0.0025632
rmse = 0.0002873

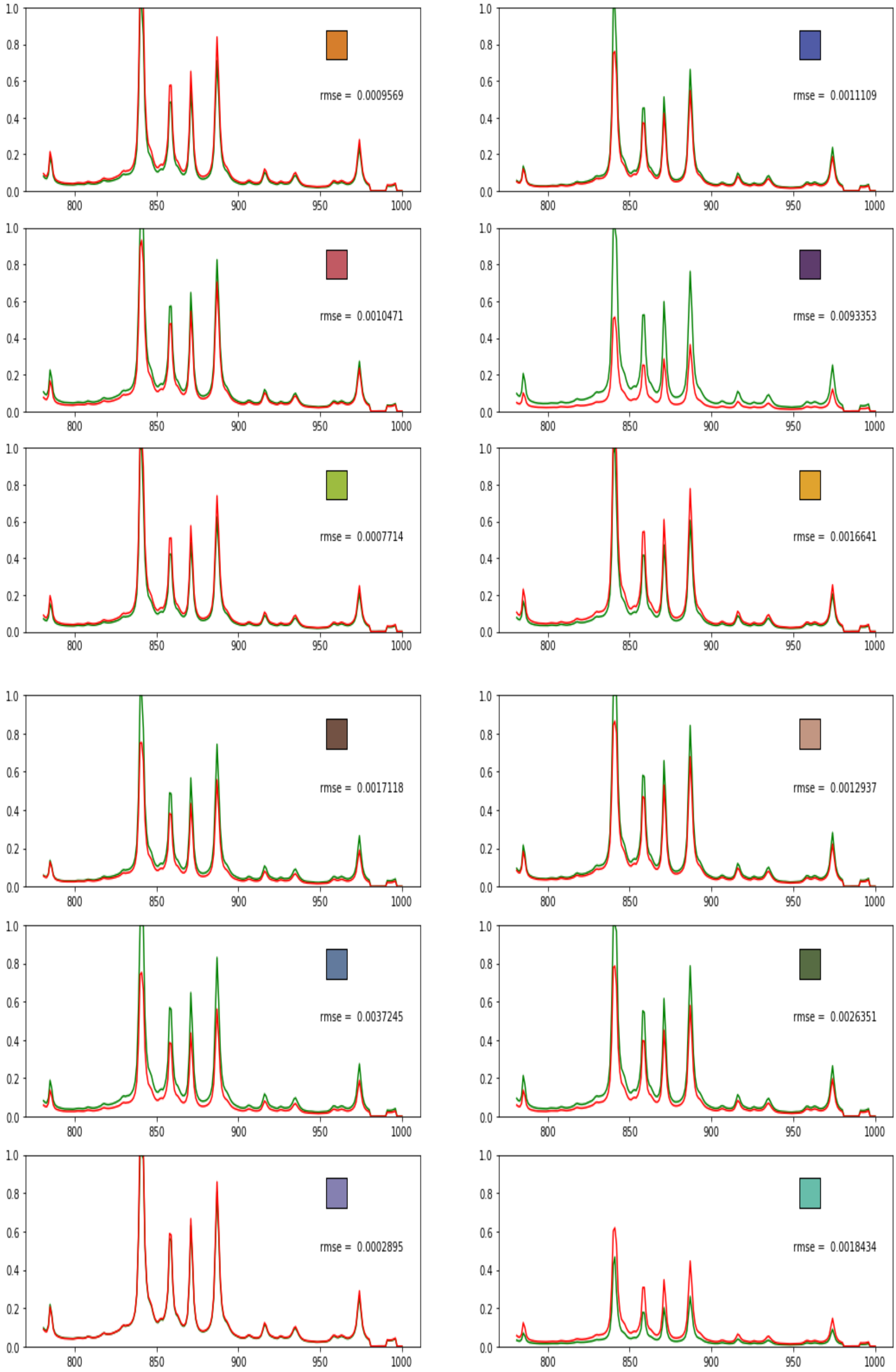

Figure S13. Near-infrared light simulation spectrum (green) and measured spectrum (red).
